# Let’s Talk About Sex: Placentas’ Central Role in Sexually Dimorphic Responses to the Maternal Milieu

**DOI:** 10.1210/clinem/dgaa683

**Published:** 2020-09-23

**Authors:** Perrie O’Tierney-Ginn

**Affiliations:** Mother Infant Research Institute, Tufts Medical Center, Boston, USA

**Keywords:** placenta, first trimester, pregnancy, sex differences, fetal growth

The higher susceptibility of male infants to nutritional deficits has been recognized for centuries. Nearly 250 years ago, obstetrician Joseph Clarke published an account of his observations of sex differences in birth outcomes based on direct measures of neonatal birth weights and head circumferences, and a birth registry of more than 20 000 cases collected at Dublin’s Lying-In Hospital. He observed that 50% more males than females were stillborn (which occurred at a rate of 1 in 20 of all births). The reason for this, he hypothesized, was that males “are naturally constituted to grow to a greater size, (thus) a greater supply of nourishment in utero will be necessary to his growth than to that of a female. Defects in this particular, proceeding from delicacy of constitution or diseases of the mother, must of course be more injurious to the male sex” (1). Indeed, his direct measures of infants at birth, some of the first published, showed that males were on average 9 oz heavier than females and had a ½-inch larger head circumference, supporting his hypothesis that males required more nutrition and may suffer more in the lack of it.

Numerous publications have supported Clarke’s findings of differences in male and female growth, his theories regarding nutritional requirements in utero (2), and males’ heightened sensitivity to maternal nutritional history (3), even among healthy pregnancies. Despite overwhelming evidence for sex differences in fetal growth and long-term outcomes, the underlying mechanism has long eluded us. In 2010, Eriksson et al reported that placentas of male fetuses are more “efficient” than those of females—a smaller placenta can support a larger fetus—but this increased efficiency comes at a cost (4). In an in utero environment with adequate nutrition, the male “lives dangerously,” his placenta performing near maximal capacity, sacrificing placental for somatic growth. The female fetus, however, displays a more conservative strategy, maintaining a larger placenta and reserve capacity, which can be drawn upon in times of poor nutrition or maternal illness. Subsequently, our group found that placental uptake of unsaturated fatty acids was lower in male, but not female, offspring of women with obesity, corresponding to changes in transporter gene expression (5). These sex differences in placental growth and nutrient handling may underlie disparities in fetal and neonatal outcomes within different nutritional environments, and originate at the molecular level.

Research on human placental sexual dimorphism has, to this point, involved full-term specimens. However, the placenta at the time of delivery is at the end of its practical life; therefore, assessment of molecular or functional alterations at this point yields few ontogenetic insights. Because early pregnancy is a critical period for fetal development, it follows that placental development may also be sensitive to the maternal milieu during the first trimester, as has been demonstrated (6). Moreover, placental adaptations in early pregnancy can define its trajectory for the remainder of pregnancy, sex-specifically altering fetal growth (7).

Persistent questions remain: How does the sex of the fetus modify maternal-placental communication throughout gestation? What are the upstream regulators that drive this crosstalk? We have, until recently, been limited to low-resolution and low-throughput assays to investigate complex molecular pathways in heterogeneous tissues such as the placenta. With the advent of single-cell RNA sequencing, the ability to assess the transcriptome in individual cell types allows perinatal biologists to tackle these questions, especially as applied to the placenta—the frontier of maternal-fetal crosstalk.

Recently, Sun et al (8) used a combination of bulk RNA sequencing and single-cell RNA sequencing to determine how fetal sex modifies the transcriptome at the maternal-placental interface in the first trimester of pregnancy. Placental villi and maternal decidua were collected at 10 to 13 weeks’ gestation from normal ongoing pregnancies (3 male and 3 female), via chorionic villous sampling, for single-cell RNA sequencing. Additionally, previously generated bulk RNA sequencing data of placental tissue collected in early pregnancy by chorionic villous sampling was used to detect differentially expressed genes (DEGs) in 17 males and 22 females. Multiple DEGs were identified between sexes in early pregnancy samples. Next, the authors took the novel step of using pathway analysis software to identify upstream regulators (eg, hormones, growth factors, mitochondrial RNA) to better understand the mechanism underlying differences between sexes. To assess the sexually dimorphic nature of maternal-fetal crosstalk, Sun et al also identified receptor-ligand pairs among the DEGs, where 1 partner was expressed in the decidua. Through the combined bulk and higher resolution single-cell analyses, Sun et al confirmed the important role of sex hormones in sexually dimorphic gene expression in trophoblasts and stromal cells. More interestingly, they found that cytokines play a key role in regulation of male and female differences in gene expression in the immune cell population of the placenta. The finding that DEGs that are upregulated in male trophoblasts are enriched in protein translation, mitochondrial and ribosomal functions is consistent with previous studies that placentas of male offspring are highly efficient and operate near maximal capacity (4). In contrast, female placentas are enriched in DEGs upregulated in cytokine-mediated signaling and response to stimuli, characteristic of a tissue on high alert for environmental stress cues, moderating growth and conserving energy-expensive metabolic pathways. The latter may explain our previous finding that although male fetal growth is highly sensitive to maternal nutritional cues, female growth outcomes are significantly altered by maternal inflammatory cytokines such as C-reactive protein and IL-6, in utero (3).

Though the study by Sun et al (8) is limited by a small sample size and a homogenous population (all were non-Hispanic white mothers), their data are novel and highly impactful to our field. They have confirmed that sexually dimorphic signaling between mother and placenta exists as early as 10 weeks of gestation. The gene pathways and upstream regulators identified by Sun et al can be validated and pursued in larger, diverse populations. We are a step closer to identifying the mechanisms underlying sex-specific fetoplacental growth, sensitivity to maternal milieu, and long-term offspring outcomes, as observed more than 200 years ago by Joseph Clarke (1).

## Data Availability

Data sharing is not applicable to this article as no datasets were generated or analyzed during the current study.

## Additional Information

***Disclosure Summary:*** No conflicts to disclose.
